# AviaTAD-LGH: A Multi-Task Spatio-Temporal Action Detector with Lightweight Gradient Harmonization for Real-Time Avian Behavior Monitoring

**DOI:** 10.3390/s26072088

**Published:** 2026-03-27

**Authors:** Zihui Xie, Haifang Jian, Wenhui Yang, Mengdi Fu, Wanting Peng, Markus Peter Eichhorn, Ramiro Daniel Crego, Xin Ning, Jun Du, Hongchang Wang

**Affiliations:** 1 School of Communication and Electronic Engineering, Shandong Normal University, Jinan 250358, China; xiezihui@semi.ac.cn (Z.X.); dujun@sdnu.edu.cn (J.D.); 2 Institute of Semiconductors, Chinese Academy of Sciences, Beijing 100083, China; jhf@semi.ac.cn (H.J.); ningxin@semi.ac.cn (X.N.); 3 Department of Landscape Architecture, College of Architecture and Urban Planning, Tongji University, 1239 Siping Road, Yangpu District, Shanghai 200092, China; 2210301@tongji.edu.cn (W.Y.); 22227@tongji.edu.cn (W.P.); 4 State Key Laboratory of Environmental Criteria and Risk Assessment, Key Laboratory of Regional Eco-Process and Function Assessment of the Ministry of Ecology and Environment, Chinese Research Academy of Environmental Sciences, Beijing 100012, China; fumd@craes.org.cn; 5 School of Biological, Earth and Environmental Sciences, University College Cork, Distillery Fields, North Mall, T23 N73K Cork, Ireland; markus.eichhorn@ucc.ie (M.P.E.); rcrego@ucc.ie (R.D.C.)

**Keywords:** video sensing, deep learning, spatio-temporal action detection, multi-task learning, ecological monitoring

## Abstract

**Highlights:**

**What are the main findings?**

- We introduce a spatio-temporal action detection benchmark for wintering cranes, featuring dense annotations for six fine-grained behaviors in long, continuous ground-camera videos under cluttered, real-world conditions.
- We propose AviaTAD-LGH, a real-time multi-task spatio-temporal action detector trained with Lightweight Gradient Harmonization (LGH) to stabilize joint optimization and improve fine-grained behavior detection. On the wintering-crane benchmark, AviaTAD-LGH reaches 68.60% mAP, surpassing strong public baselines. Compared with a single-task variant, it improves mAP by 2.80 percentage points and boosts AP by more than 12 percentage points on challenging classes.

**What are the implications of the main findings?**

- AviaTAD-LGH enables efficient, individual-level behavior localization and recognition from continuous monitoring streams, supporting scalable video-based ecological monitoring and restoration assessment.
- LGH is a plug-and-play training strategy for multi-task video understanding, improving optimization without adding inference-time complexity and remaining suitable for edge-oriented real-time sensing applications.

**Abstract:**

Fine-grained spatio-temporal action detection in continuous, unconstrained field videos remains a formidable challenge due to severe background clutter, high inter-class similarity, and the scarcity of domain-specific benchmarks. To address these limitations, we first introduce a large-scale Wintering-Crane Benchmark, providing dense, individual-level bounding box annotations for six complex behaviors across diverse habitat scenes. Leveraging this data, we propose AviaTAD-LGH, a real-time multi-task framework that incorporates auxiliary motion supervision into a dual-pathway 3D backbone to enhance feature discriminability. A critical bottleneck in such multi-task settings is the negative transfer caused by conflicting optimization objectives. To resolve this, we present Lightweight Gradient Harmonization (LGH), a plug-and-play optimization strategy that dynamically modulates task weights based on the cosine similarity of gradient directions. This mechanism effectively aligns optimization trajectories without introducing inference latency. Extensive experiments demonstrate that AviaTAD-LGH achieves a state-of-the-art mAP of 68.60%, surpassing strong public baselines by 7.44% and improving upon the single-task baseline by 2.80%, with significant gains observed on ambiguous dynamic classes. The proposed pipeline enables efficient, scalable ecological monitoring suitable for edge deployment.

## 1. Introduction

Habitat fragmentation, climate change, and unsustainable human activities have led to a continuous decline in global biodiversity, becoming a major global concern [1,2]. In response to habitat changes, many species adjust their behaviours—such as habitat selection and foraging strategies—to maintain or improve individual fitness. For example, the Oriental stork shows notable shifts in habitat use and foraging strategies between breeding sites, migratory stopovers, and wintering grounds [3]. Continuous monitoring and quantitative analysis of animal behaviour are essential for understanding species’ activity patterns and habitat use, thus providing critical data for wildlife conservation and ecosystem management [4].

In behavioural ecology, time allocation to different activities is a widely used metric for quantifying behaviours and assessing their relative importance [5]. Traditional animal behaviour studies have relied heavily on direct human observation to record the occurrence and duration of specific behaviours. With advances in imaging and sensing technologies, video-based devices have become essential tools for indirect behavioural observation. In recent years, infrared cameras, acoustic monitoring systems, and drones have significantly improved the spatio-temporal resolution and coverage of field data collection [6,7]. Despite these advances, large volumes of video data still lack automated and quantitative analysis frameworks, and the application of efficient computer vision and deep learning models in animal behaviour research remains limited [8,9].

Existing methods mostly focus on species detection or single-frame posture classification from static images, thereby assigning a “behaviour-at-this-moment” label to isolated frames. However, this approach inherently weakens the sequential nature of behaviour and makes it difficult to capture complete behavioural episodes, thereby hindering real-time monitoring of continuous video streams in conservation practices. To support conservation and management decisions, practical systems need to operate on long, continuous field videos and provide reliable spatio-temporal localization and recognition at the individual level.

In recent decades, China has experienced a significant reduction in the area of natural wetlands, along with pronounced changes in wetland types—large areas of natural lakes, marshes, and intertidal wetlands have been converted into farmland, reclaimed, or transformed into artificial ponds and non-wetland land uses [10,11]. Waterbirds, particularly cranes, serve as sensitive indicators of wetland ecosystem health due to their dependence on wetland habitats and sensitivity to disturbance [12,13]. Of the 15 extant crane species, nine are recorded in China, with eight being migratory [14]. Their foraging and activity patterns directly reflect food availability and habitat quality [15], making behavioural monitoring valuable for evaluating wetland restoration outcomes [16,17]. As such, deploying intelligent video analysis on compact AI platforms has shown promise in various real-time sensing applications, including trajectory prediction and task offloading in IoT systems [18].

**Figure 1:**
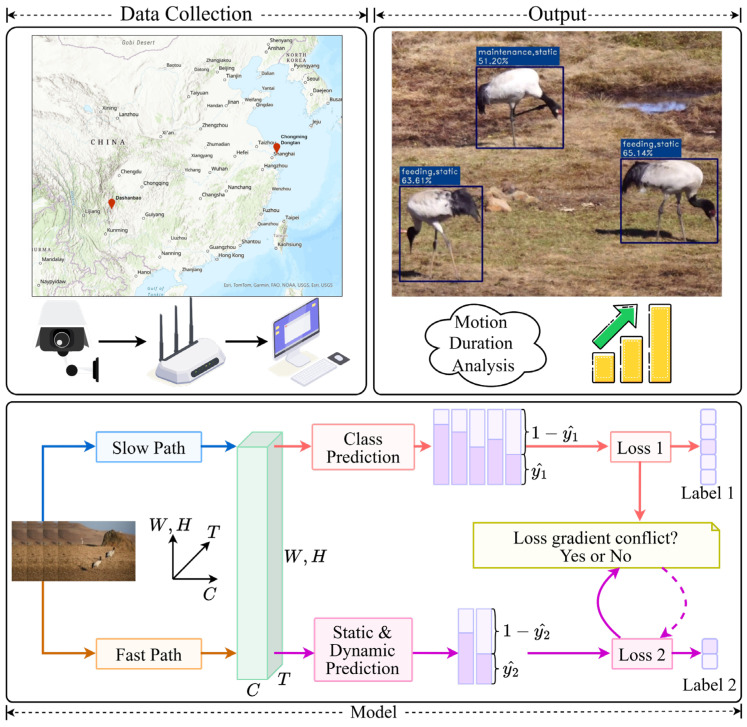
AviaTAD-LGH system architecture diagram.

Motivated by these gaps, we develop a real-time deep learning-based spatio-temporal action detection framework for continuous crane behaviour monitoring from ground-based video streams and introduce a lightweight gradient coordination strategy to improve fine-grained behaviour recognition by leveraging auxiliary supervision during training (Figure 1).

Our main contributions are threefold:

A spatio-temporal action detection benchmark for crane behavior: We curate and annotate a large-scale video dataset of wintering crane behavior using infrared camera footage from nature reserves. The dataset covers multiple habitats, including forests and wetlands, and includes six fine-grained behaviors: feeding, vigilance, social behavior, maintenance, walking, and flight. We provide frame-level bounding boxes and static/dynamic motion-attribute labels. Additionally, we systematically analyze the joint distribution of behavior and motion types, as well as the geometric characteristics of detection boxes, establishing a representative spatio-temporal multi-task benchmark for waterbird behavior recognition.

AviaTAD-LGH: a multi-task spatio-temporal action detector with LGH gradient coordination: This involves a dual-branch multi-task architecture that simultaneously predicts behavior categories and motion attributes. We introduce a Lightweight Gradient Harmonization (LGH) strategy, which adaptively adjusts task loss weights based on the cosine similarity of task gradients. This alleviates negative transfer during multi-task training without increasing model parameters, enabling a better balance between fine-grained behavior recognition and static/dynamic state classification. The overall design is simple and can be seamlessly integrated into existing video models.

State-of-the-Art Performance and Real-World Deployment: We evaluate the model on a compact AI platform (NVIDIA DGX Spark) and confirm that AviaTAD-LGH meets real-time inference requirements (≥25 fps) under a 240 W power envelope. By combining model outputs with time-budget analysis, diversity indices, and principal component analysis, we quantitatively assess the habitat quality of wintering crane grounds, demonstrating AviaTAD-LGH’s practical potential for real-world wetland management. By combining model outputs with time-budget analysis, diversity indices, and principal component analysis, we quantitatively assess the habitat quality of wintering crane grounds, demonstrating AviaTAD-LGH’s practical potential for real-world wetland management. Although validated on wintering crane behavior, the proposed multi-task spatio-temporal framework and LGH strategy are species-agnostic by design. In principle, they can be transferred to other wildlife video behavior tasks, provided that appropriate behavior labels and datasets are available for the target species.

## 2. Related Work

### 2.1. Datasets and Ecological Monitoring

To advance automated behaviour monitoring from continuous field videos, recent large-scale animal video benchmarks such as MammalNet provide important training and evaluation resources, but they are not tailored to wetland bird behaviours and habitat-specific monitoring needs [19]. Beyond video benchmarks, camera-trap and citizen-science initiatives have released influential wildlife datasets that accelerate visual recognition in ecology, such as the iWildCam competition dataset for cross-location generalization in camera-trap imagery [20] and Snapshot Serengeti with high-frequency annotations collected from long-term deployments [21]. These resources have also inspired scalable automated pipelines for species identification and ecological data extraction using deep learning [22]. In parallel, pose- and behaviour-quantification toolkits (e.g., markerless pose estimation) have broadened the methodological toolbox for animal behaviour analysis from videos, especially when labels are scarce [23]. Overall, while existing datasets and tools have greatly improved wildlife recognition and ecological monitoring, they typically provide image-level labels or coarse event tags and rarely support individual-level spatio-temporal localization of fine-grained wetland-bird behaviours in long, cluttered field streams—thereby motivating a crane-focused benchmark with explicit spatio-temporal annotations for continuous habitat monitoring.

### 2.2. Video Action Recognition

In human-centric action understanding, widely used benchmarks such as Kinetics and AVA have driven progress in video recognition and spatio-temporal action localization, offering methodological foundations for transferring to wildlife scenarios [24,25]. Early clip-based video classification pipelines typically rely on short-window aggregation and are therefore insufficient for capturing full behavioural episodes in long, continuous streams [26]. Two-Stream CNN improves motion sensitivity by jointly modelling appearance and optical flow, yet it still often operates on bounded clips rather than temporally complete behaviour segments [27]. To learn richer spatio-temporal representations, 3D convolutional architectures such as C3D and I3D perform end-to-end feature learning over video volumes and have become standard backbones for video understanding [28,29]. SlowFast further strengthens motion modelling via dual pathways with different frame rates, achieving strong performance on standard benchmarks and motivating its adaptation to real-world monitoring settings [30]. More recently, transformer-based video backbones have shown strong performance by modelling long-range dependencies, including TimeSformer [31], Multiscale Vision Transformers (MViT) [32], and locality-biased designs such as Video Swin Transformer [33]; self-supervised pretraining further reduces reliance on large, labelled video corpora in downstream tasks. In summary, although modern backbones are increasingly powerful, many are still developed and evaluated under trimmed-clip or offline assumptions, and their speed–accuracy trade-offs and robustness for continuous, real-time spatio-temporal detection in field surveillance (with strong background clutter and long untrimmed streams) remain insufficiently characterized—thus motivating our deployment-oriented AviaTAD-LGH design built for real-time, individual-level monitoring. In practice, real-time monitoring on edge devices often relies on model compression and mixed-precision optimization to improve the speed–accuracy trade-off for video models [34].

### 2.3. Multi-Task Learning and Gradient Conflicts

Despite these advances, wildlife behaviour datasets are often small and imbalanced, making it necessary to leverage pretraining and transfer learning to adapt video models to domain-specific field scenes [35]. Multi-task learning provides a principled way to jointly optimize related objectives in a shared representation space, improving data efficiency and generalization for complex behaviour understanding tasks [36]. However, multi-task optimization may suffer from negative transfer when task gradients conflict, which can degrade the primary task performance if not handled properly [37]. To address task imbalance and instability, adaptive loss weighting methods such as GradNorm and uncertainty-based weighting have been proposed to dynamically balance objectives during training [38,39]. More recent work further explores consistency-driven task specialization and expert-based mechanisms to mitigate gradient conflicts and stabilize multi-task learning, inspiring lightweight coordination strategies for practical deployment [40,41]. Beyond gradient projection and normalization, alternative perspectives on gradient dynamics have also been explored, such as the Focal Quotient Gradient System method [42], which provides a complementary lens on gradient-based training of deep neural networks. In addition, game-theoretic gradient combination has been proposed to produce principled multi-task update directions (e.g., Nash-MTL) [43]. Taken together, existing MTL strategies highlight the importance of conflict mitigation, but many solutions introduce extra complexity (additional modules/experts or heavier optimization), leaving room for a lighter plug-in coordination that resolves gradient conflicts without adding parameters—this directly motivates our Lightweight Gradient Harmonization (LGH) for jointly learning behaviour detection and motion attributes.

## 3. Materials and Methods

### 3.1. Dataset Collection and Annotation

To support this study, we constructed a large-scale spatio-temporal action dataset for bird behaviour analysis. Source videos were collected from infrared camera footage provided by collaborating nature reserves. Field videos were acquired using fixed ground-based infrared cameras deployed for routine ecological surveillance. Cameras were installed at stable viewpoints to provide continuous, non-invasive observations of crane activities. Videos were recorded with embedded timestamps and then segmented into short clips for annotation and model development. To protect sensitive conservation sites, exact camera coordinates and deployment details were anonymized during data release and analysis. These videos cover crane activities across multiple representative habitat types, such as forests and wetlands. The dataset includes footage from diverse monitoring sites and temporal periods, enhancing the model’s generalization across different scenes. Cranes were selected as the focal species due to their moderate size and rich behavioural repertoire, including flight, foraging, and social interaction. In total, we collected 200 raw video sequences, each approximately 1800 s (30 min) in duration, amounting to approximately 100 h of footage. Videos were recorded at 1920 × 1080 resolution and 30 fps using fixed ground-based infrared cameras deployed for routine ecological surveillance. From these raw sequences, we extracted keyframes at regular intervals and annotated all visible crane individuals on each keyframe, yielding 19,926 spatio-temporal instance annotations (i.e., individual-level bounding-box annotations, each associated with a keyframe timestamp, a behavior label, and a motion-attribute label). Because multiple cranes are often visible in the same keyframe, the number of instance annotations substantially exceeds the number of unique keyframe timestamps. During training and inference, for each keyframe annotation, the data loader samples a temporal context window of T consecutive frames centered on that keyframe, and each individual’s bounding box is processed via ROI Align to extract its spatio-temporal features for classification.

We collaborated with ornithology experts to define the behavioral taxonomy and annotation protocol (Table 1). All bounding-box annotations and behavioral labels were produced manually using the VGG Image Annotator (VIA) tool, following the spatio-temporal annotation pipeline described in the open-source AVA-Dataset framework. The annotation procedure consisted of four steps: (1) extracting keyframes from each video clip at the target frame rate; (2) annotators manually drawing bounding boxes around each visible crane individual and assigning both behavior-category and static/dynamic motion-attribute labels on each keyframe using the VIA tool; (3) the manually annotated bounding boxes were fed into the DeepSORT tracking algorithm to associate the same individual across consecutive frames, producing temporally coherent individual IDs; (4) all ID assignments were subsequently reviewed and corrected manually to ensure temporal consistency, followed by format conversion to AVA-style CSV files using the AVA-Dataset scripts. The final taxonomy comprises six fine-grained behavior categories: feeding, vigilance, social behavior, maintenance, walking, and flight.

These six behaviors encompass the main aspects of birds’ daily activities. To leverage motion differences between behaviors, each individual bounding-box annotation is assigned a binary motion attribute. An individual instance is labeled as static (0) when the bird’s position changes little, and its movement amplitude is limited within the annotated temporal window; conversely, instances showing significant displacement or large body movements are labeled as dynamic (1). Importantly, this label is assigned at the individual bounding-box level, not at the clip level: different individuals within the same frame may receive different motion-attribute labels depending on their respective movement patterns. This attribute does not directly correspond to specific behavior categories, as both static and dynamic instances may occur within the same class. Annotators considered displacement, speed, and movement amplitude when determining the motion state. When multiple cranes appear simultaneously in a video, each individual is assigned a unique integer ID within the video sequence to enable temporal tracking. All bounding boxes were drawn manually by annotators using the VIA tool. The manually annotated boxes were then fed into the DeepSORT tracking algorithm to associate the same individual across consecutive frames, producing temporally coherent IDs. The tracking outputs were subsequently reviewed and corrected manually to ensure ID consistency. All annotations are stored in CSV format, with each row recording a single spatio-temporal instance—defined as one bounding-box annotation for one individual at one timestamp—including video ID, frame ID, individual ID, behavior category ID, motion attribute label, bounding-box coordinates, and other metadata. The dataset is split at the video level into training, validation, and test sets with an 8:1:1 ratio.

The final dataset contains 19,926 annotated spatio-temporal instances. Following an 8:1:1 split at the video level, the dataset is divided into 15,941 instances for training, 1993 for validation, and 1992 for testing. In the dataset, the proportions of the six behavior classes are as follows: Maintenance (33.7%), Feeding (31.3%), Vigilance (18.8%), Walk (6.6%), Flight (6.3%), and Social Behavior (3.3%). Static behaviors account for 70.6%, while dynamic behaviors make up 29.4%, a distribution that accurately reflects the natural activity budget of the observed species. High-frequency routine behaviors, such as maintenance and feeding, are well-represented, whereas more intermittent and specialized behaviors, like social interaction and flight, have fewer samples. This imbalance aligns with ecological expectations where birds spend the majority of their time in energy-conserving or foraging states. To ensure model robustness, the class-wise and static/dynamic proportions across the training, validation, and testing sets are maintained with a variance of less than 2%, minimizing the risk of the model overfitting to specific video-level behavioral patterns.

To ensure annotation quality, three PhD students with ornithological training performed the initial labeling under a unified protocol, and an expert with years of field-monitoring experience conducted review and corrections. The labeling process consisted of three steps: (1) preliminary annotation of candidate clips based on behavioral definitions, (2) group discussion and standardization for samples with disagreements, and (3) random sampling for re-checking to assess overall consistency. To quantify annotation consistency, we randomly sampled 500 spatio-temporal instances and had all three annotators independently re-label them. The mean pairwise IoU for bounding-box localization was 0.85 (±0.07). For behavior-category labels, the mean pairwise Cohen’s kappa was 0.89 (near-perfect agreement). For the binary motion attribute, the mean pairwise Cohen’s kappa was 0.91. The main sources of disagreement were: (i) the feeding–vigilance boundary, where brief head-raising episodes (<1 s) during continuous foraging were ambiguous; and (ii) the walking–feeding boundary, where slow locomotion with intermittent pecking could be assigned to either class. To resolve these hard cases, we adopted explicit decision rules: head-raising shorter than 1 s during an otherwise continuous foraging bout is labeled as feeding; slow movement accompanied by continuous pecking is labeled as feeding rather than walking. These rules were documented in the annotation protocol and applied consistently during expert review.

**Figure 2:**
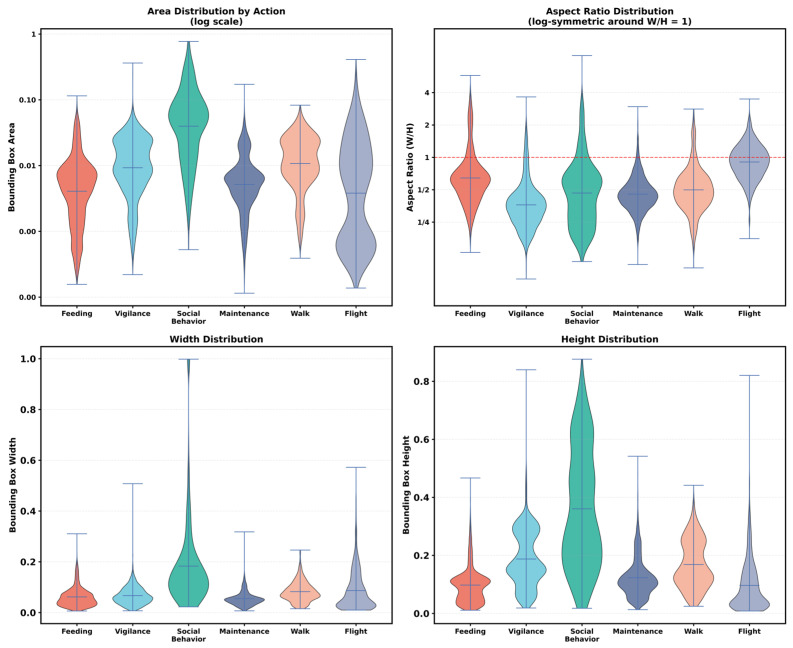
Bounding-box feature analysis.

Figure 2 summarizes the geometry of detection boxes across behaviours and reveals pronounced inter-class heterogeneity in scale and shape. To improve the visibility of small boxes and the symmetry of width-to-height relationships, the area distribution is shown on a logarithmic scale and the aspect ratio distribution is displayed on a log-symmetric scale around W/H = 1. Social Behavior stands out with substantially larger and more variable boxes, showing the broadest spread in area and the widest aspect ratio range, with widths and heights occasionally spanning a large fraction of the frame, reflecting frequent group-level scenes. In contrast, the remaining behaviours are dominated by small, single-bird boxes with mostly near-square aspect ratios and relatively concentrated width distributions. Among these single-bird actions, Vigilance tends to produce relatively taller boxes, whereas Feeding yields shorter boxes. Overall, these statistics indicate strong variation in object scale and box geometry across behaviours, which increases the difficulty of learning a unified detector under continuous field monitoring conditions.

All videos used in this study were collected as part of routine infrared monitoring by collaborating nature reserves. Camera deployment followed relevant laws, regulations, and reserve management guidelines, ensuring minimal disturbance to the birds and their habitats. No additional external video sources were used. Sensitive geographic information was anonymized during data processing and release.

### 3.2. AviaTAD-LGH Model Architecture

**Figure 3:**
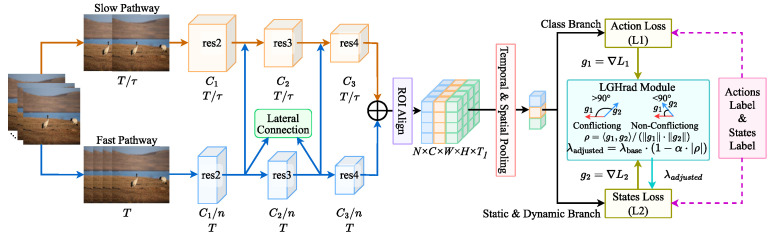
AviaTAD-LGH model architecture diagram.

AviaTAD-LGH employs a 3D-convolution-based video backbone for spatio-temporal feature extraction and uses a dual-branch multi-task architecture (Figure 3). The system consists of a shared spatio-temporal feature extractor and two parallel classification heads: one for the primary task of behavior classification and the other for the auxiliary task of motion-attribute prediction. The input is a video clip of T = 32 frames, which undergoes standard preprocessing before being fed into a 3D ResNet-50-style backbone. The backbone follows a dual-frame-rate design: a slow pathway samples one frame every 8 frames to capture stable appearance and scene semantics, while a fast pathway samples one frame every 2 frames to capture short-term motion dynamics. The two pathways are connected laterally at each residual stage, enabling feature exchange and fusion, ultimately generating spatio-temporal feature maps that jointly encode appearance and motion information.

On the left, the input video is processed by a slow pathway (sparse sampling) and a fast pathway (dense sampling), which extract static appearance cues and dynamic motion cues, respectively. The backbone fuses information from the two pathways to produce spatio-temporal feature maps. An ROI Align module then extracts features from the target regions and applies spatio-temporal pooling. On the right, the shared features are passed through two sets of fully connected layers to generate behavior category predictions (primary task) and motion-attribute predictions (auxiliary task).

Based on the spatial locations of the birds, the model extracts target-region features via a spatio-temporal extension of ROI Align. Following the SlowFast design [30], the slow pathway (C_slow = 2048) and fast pathway (C_fast = 256, with channel_ratio = 8) feature maps are first concatenated along the channel dimension after the final residual stage, yielding a unified 3D feature volume **F** of shape 2304 × T × H × W. ROI Align is then applied on this concatenated volume: for each bounding-box proposal, a spatially fixed-size (8 × 8) feature crop is extracted at every temporal position, producing a per-proposal tensor of shape 2304 × T × 8 × 8. This implementation follows the SingleRoIExtractor3D module with temporal pooling enabled. Spatio-temporal pooling is subsequently applied in two stages: temporal average pooling reduces the time dimension to 1, and spatial max pooling collapses the 8 × 8 spatial grid to 1 × 1. The result is a D-dimensional (D = 2304) global feature vector **f** per proposal, encoding the aggregated spatio-temporal representation of the target individual over T frames. This feature vector is denoted as

(1) f=Pool(ROIAlign(F,b)),

where F denotes the spatio-temporal feature maps produced by the backbone network and b denotes the bounding-box coordinates.

The shared feature f is then fed in parallel into two classification branches. The behavior classification branch (primary task) maps f through a fully connected layer into a 6-dimensional behavior space. The classification head produces a 6-dimensional logit vector, which is passed through a sigmoid function to obtain per-class probabilities. Training of this branch uses a multi-label binary cross-entropy loss:

(2) $$L_{\mathrm{act}}=-\frac{1}{N}\sum_{i=1}^{N}\sum_{c=6}^{C}\left[y_{i,c}\log(p_{i,c})+(1-y_{i,c})\log(1-p_{i,c})\right],$$

where N is the number of samples, C is the number of behavior classes, y denotes the ground-truth labels and p the predicted probabilities.

The motion-attribute branch is a two-layer MLP: a fully connected hidden layer of dimension D with ReLU activation, followed by a 2-dimensional linear output layer. During training, the output logits are passed to the standard cross-entropy loss; during inference, softmax converts them to static/dynamic probabilities. This branch is trained with the standard cross-entropy loss:

(3) $$L_{\mathrm{mov}}=-\frac{1}{N}\sum_{i=1}^{N}\sum_{m=0}^{1}y_{i,m}\log(p_{i,m}),$$

where m indicates the static or dynamic state.

The two branches share all parameters of the backbone network, and only the parameters of the classification layers are task-specific. This design preserves diversity at the decision level while maintaining joint learning of low- and mid-level features. By enforcing a shared feature space, the model is encouraged to learn representations that simultaneously support fine-grained behavior recognition and discrimination of motion magnitude.

In a multi-task learning framework, interference between loss gradients of different tasks becomes a central optimisation challenge. When the gradients of the primary and auxiliary tasks are aligned, joint training yields synergistic effects; however, when they form a large angle, naïvely summing the gradients can drive each task away from its optimal optimisation trajectory.

We address this gradient conflict problem with the proposed LGH mechanism. The core idea of LGH is to monitor the directional relationship between task gradients and adaptively adjust the loss weights, steering parameter updates along more favourable directions. Formally, let $L_{\mathrm{act}}$ denote the behavior classification loss, $L_{\mathrm{mov}}$ the motion-attribute loss, and θ the model parameters. Following standard practice in multi-task gradient analysis, we compute the gradient cosine similarity over the shared backbone parameters θ_shared only, excluding the task-specific classification heads. This is because gradient conflicts are most impactful at the shared parameters that must simultaneously serve both tasks; the task-specific heads receive single-task gradients and are therefore free from inter-task interference. At each mini-batch optimisation step, we compute the gradients of each task with respect to θ:

(4) $$g_{\mathrm{act}}=\nabla_{\theta }L_{\mathrm{act}},\;g_{\mathrm{mov}}=\nabla_{\theta }L_{\mathrm{mov}}.$$

The cosine similarity between the two gradients is defined as

(5) $$\rho =\cos(g_{\mathrm{act}},g_{\mathrm{mov}})=\frac{g_{\mathrm{act}}\cdot g_{\mathrm{mov}}}{\left|g_{\mathrm{act}}||g_{\mathrm{mov}}\right|}.$$

This metric quantifies the alignment of the gradients. A positive value indicates agreement, meaning the gradients reinforce each other, while a negative value signals a conflict, where the gradients oppose each other, potentially hindering the optimization process.

LGH dynamically adjusts the auxiliary-task weight based on the cosine similarity between task gradients. We set a conflict reduction factor α ∈ [0, 1] and a base weight $\lambda_{\mathrm{base}}$ > 0. When α = 0, LGH reduces to fixed weighting; when α = 1, the auxiliary weight is maximally suppressed under full conflict. The auxiliary task’s influence is reduced when a conflict is detected when gradients are misaligned, and restored when gradients align. The weight adjustment follows the formula:

(6) $$\lambda =\left\{\begin{array}{l} \lambda_{\mathrm{base}}\cdot (1-\alpha \cdot |\rho |),\;if\rho <0,\\ \lambda_{\mathrm{base}},\;\mathrm{otherwise}. \end{array}\right.$$

where ρ represents the cosine similarity between the gradients of the two tasks. This dynamic scaling ensures that the auxiliary task’s influence is modulated based on how misaligned the task gradients are.

This dynamic modulation mechanism adjusts the auxiliary task weight based on the cosine similarity between the task gradients. When a gradient conflict is detected, the weight is reduced. As the gradients align and become cooperative, the weight is gradually restored to its standard value. The overall loss is given by

(7) $$L_{\mathrm{total}}=L_{\mathrm{act}}+\lambda \cdot L_{\mathrm{mov}}.$$

LGH ensures that the combined gradient $g_{\mathrm{total}}=g_{\mathrm{act}}+\lambda \cdot g_{\mathrm{mov}}$ is constrained within a cone centred around the primary-task gradient, preventing the update direction from deviating excessively from the main optimisation objective. Unlike explicit gradient-projection methods such as Projected Conflicting Gradient (PCGrad), LGH does not directly modify the gradient vectors; instead, it imposes an indirect constraint in the loss space by rescaling the auxiliary-task loss. It also differs from adaptive loss-weighting schemes such as Gradient Normalization (GradNorm), which require additional network branches or complex normalisation of task gradients. LGH introduces only a single scalar weight, making it easy to integrate into existing video recognition backbones.

## 4. Results

### 4.1. Model Training and Validation

We use SGD as the optimiser with an initial learning rate of 0.2 and a weight decay of $1\times 10^{-5}$. Training is carried out on eight NVIDIA RTX 4090 GPUs (NVIDIA Corporation, Santa Clara, CA, USA), with a batch size of 2 per GPU and a total batch size of 16.

The primary evaluation metric is mean Average Precision (mAP). For each behavior category, we compute the Average Precision (AP) by ranking all clips according to the model’s detection confidence and integrating the area under the corresponding Precision-Recall curve. The mAP is obtained by averaging the AP across the six behavior classes.

### 4.2. Main Results and Analysis

We compare AviaTAD-LGH with several baseline models on our dataset to validate the effectiveness of the proposed approach. Table 2 summarizes the overall mAP and per-class AP for all methods. The baseline model (single-task, without the auxiliary head) achieves an mAP of 65.80%. ACRNN-SlowFast integrates an Attention-augmented Convolutional Recurrent Neural Network with the SlowFast backbone. It augments the spatial features with a channel-attention mechanism and models temporal dependencies through a convolutional LSTM module, thereby enhancing the capacity for sequential behavior recognition. ACRNN-SlowFast achieves 61.43%. VideoMAE-Base, used as a reference, achieves 61.16% [44]. VideoMAE-Large, a larger model with unsupervised pre-training, reaches 67.60%, which is still slightly below our multi-task AviaTAD-LGH model. This suggests that task-specific optimization (multi-task learning with LGH) is more beneficial than increasing model capacity alone.

The full AviaTAD-LGH model achieves an mAP of 68.60%, improving upon the baseline by 2.80 percentage points (Table 2). At the per-class level, AviaTAD-LGH achieves 83.18% AP on Feeding (baseline: 75.78%), 54.77% on Vigilance (baseline: 41.19%), and 23.20% on Walk (baseline: 10.43%). These gains indicate that auxiliary learning of motion attributes significantly helps the model better recognize these classes: vigilance is largely static with subtle visual differences, and the multi-task model captures these fine-grained cues; feeding involves both static and dynamic instances, and the multi-task framework effectively exploits this prior knowledge.

To assess the contribution of the auxiliary motion task and different multi-task optimization strategies, the results are summarized in Table 3. The single-task baseline achieves 65.80% mAP. We then evaluate representative multi-task optimization methods that either rebalance task contributions during training or explicitly mitigate gradient conflicts between the behaviour and motion tasks. GradNorm produces a modest gain (66.21% mAP, +0.41 pp), indicating that task-imbalance exists but is not the dominant bottleneck in this setting. In contrast, conflict-aware gradient methods lead to larger improvements. PCGrad increases mAP to 66.92% (+1.12 pp) by projecting away conflicting components in task gradients, which partially alleviates negative transfer but may be sensitive to noisy gradient estimates in small, long-tailed field datasets. RI-PCGrad [45] extends the original PCGrad by incorporating gradient rescaling to control the relative influence of each task after projection, achieving improved stability over the base method. Unlike both PCGrad and RI-PCGrad, which explicitly modify gradient vectors, LGH imposes an indirect constraint in the loss space by rescaling the auxiliary-task loss, avoiding the computational overhead of per-parameter gradient manipulation. RI-PCGrad further improves performance to 67.25% (+1.45 pp) by incorporating rescaling to better control the relative influence of tasks, suggesting that both gradient direction and magnitude matter when optimizing fine-grained behaviours with an auxiliary motion cue. Finally, our LGH obtains the best performance (68.60% mAP, +2.80 pp). Unlike gradient-surgery methods that modify gradients explicitly, LGH uses a lightweight, conflict-aware reweighting rule driven by the cosine similarity between task gradients, suppressing the auxiliary loss only when it would steer updates away from the primary objective. This design appears particularly effective for our continuous video setting, where the motion attribute is helpful but intermittently conflicting with behaviour classification. Overall, the results confirm that explicit interference control is crucial, and that a simple conflict-aware weighting strategy can be more robust than gradient-manipulation baselines on this dataset.

To quantify the contribution of the auxiliary motion attribute and the effect of different task-weighting schemes, we conduct a component ablation study under the same backbone and training protocol. As reported in Table 4, the single-task baseline (behaviour detection only) achieves 65.80% mAP. Introducing the auxiliary motion head without conflict handling yields a small but consistent improvement to 66.01% mAP (+0.21 pp), indicating that the motion attribute provides additional supervision for fine-grained behaviour recognition. When applying a fixed static weighting between the behaviour and motion losses (λ = 0.5), performance further increases to 67.20% mAP (+1.40 pp), suggesting that an appropriate balance between tasks is important for learning robust spatio-temporal representations. Uncertainty-based weighting [39] learns per-task homoscedastic uncertainty parameters as trainable scalars, which are used to automatically balance the relative loss contributions of different tasks during multi-task optimization; tasks with higher intrinsic uncertainty receive lower weight, and vice versa. Uncertainty-based weighting improves mAP to 67.76% (+1.96 pp), implying that generic adaptive weighting can mitigate task-imbalance effects caused by heterogeneous and long-tailed field data. Notably, this uncertainty-driven scheme adjusts the task weights according to the overall noise/scale of each objective, but it does not explicitly account for whether the two tasks produce conflicting gradient directions on shared parameters at a given step. Finally, our conflict-aware LGH achieves the best performance of 68.60% mAP (+2.80 pp) by monitoring the cosine similarity between task gradients and down-weighting the auxiliary motion loss only when it would steer updates away from the primary behaviour objective, which is more effective than static or generic adaptive weighting for continuous, cluttered wetland surveillance videos.

Table 5 reports the validation mAP under different combinations of the conflict reduction factor α and the base auxiliary weight λ_base. The best performance (68.60%) is achieved at α = 0.2, λ_base = 0.10. Performance is robust within α ∈ [0.1, 0.3], where mAP varies by less than 0.5 percentage points for a given λ_base. Increasing α to 0.5 leads to a noticeable decline, as overly aggressive conflict suppression diminishes the auxiliary task’s contribution. Similarly, both very small (0.02) and very large (0.20) values of λ_base degrade performance, indicating that an appropriate balance between auxiliary supervision strength and conflict tolerance is important. Based on these results, we adopt α = 0.2 and λ_base = 0.10 for all experiments.

Table 6 compares the complexity and inference speed of all evaluated models. All measurements are conducted on a single NVIDIA RTX 4090 GPU (NVIDIA Corporation, Santa Clara, CA, USA) with input resolution 1920 × 1080, batch size 1.

### 4.3. Practical Monitoring Evaluation

To evaluate the practical utility of AviaTAD-LGH in real-world ecological monitoring, we compare its predictions with conventional manual annotation along three dimensions: processing efficiency, detection coverage and individual-level data granularity. Two representative field-monitoring videos are used as test samples: Video 1 contains 14 individuals and Video 2 contains 12 individuals, both encompassing multiple behavioral sequences.

**Figure 4:**
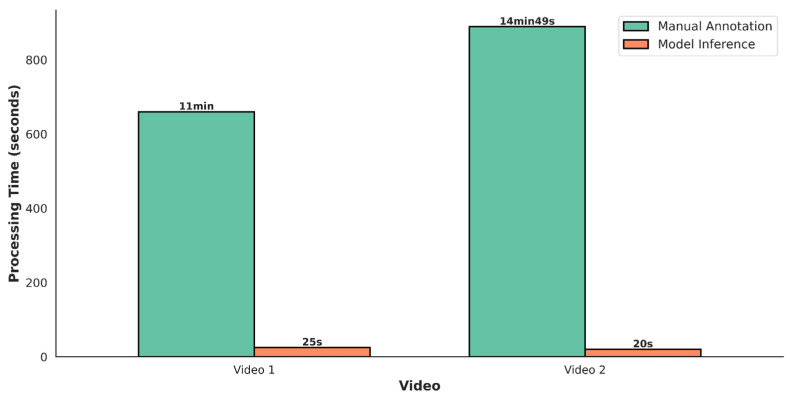
Comparison of processing time between manual annotation and AviaTAD-LGH inference.

Figure 4 contrasts the time required for manual annotation and model inference. We define the efficiency ratio as the manual annotation time divided by the model inference time for the same video. For Video 1 (total duration ≈ 30 s, with 203.6 s of accumulated behavioral time across individuals), an experienced observer requires 11 min (660 s) to complete frame-by-frame annotation, whereas AviaTAD-LGH finishes inference in 25 s, yielding an efficiency ratio of 26.4×. For Video 2 (total duration ≈ 30 s, 171.4 s of accumulated behavioral time), manual annotation takes 14 min 49 s (889 s), while the model requires only 20 s, an efficiency ratio of 44.5×. Averaging the two videos gives a mean efficiency ratio of 35.4×.

Figure 4 compares the processing time for manual annotation and model inference. The green bars represent manual annotation time (in minutes), while the orange bars show model inference time (in seconds), highlighting the significant efficiency advantage of the automated system for large-scale monitoring.

This efficiency gain is crucial for long-term ecological monitoring. To illustrate, consider a typical wetland reserve collecting 10 h of surveillance video per day. Based on our measured efficiency ratios, manual frame-by-frame annotation would require approximately 10 × 35.4 = 354 person-hours per day—clearly infeasible for routine monitoring. In contrast, AviaTAD-LGH can process the same footage in approximately 10 × 60/35.4 ≈ 17 min of GPU compute time, reducing human effort to targeted auditing of model outputs and manual inspection of low-confidence or anomalous events.

To further validate deployment feasibility, we exported the trained AviaTAD-LGH model to TensorRT FP16 format and evaluated inference on the NVIDIA DGX Spark, a compact desktop AI platform (150 × 150 × 50.5 mm, 1.2 kg) powered by the GB10 Grace Blackwell Superchip. DGX Spark integrates a Blackwell GPU with fifth-generation Tensor Cores and a 20-core ARM Grace CPU, providing 128 GB of unified LPDDR5x memory (273 GB/s bandwidth) and up to 1 PFLOP of FP4 AI compute at a peak system power of 240 W. Under realistic monitoring conditions (1920 × 1080 input, batch size 1), AviaTAD-LGH achieves an end-to-end throughput of approximately 28 fps with a per-clip latency of ~36 ms and an inference power draw of ~170 W. These results confirm that AviaTAD-LGH meets the real-time requirement of ≥25 fps on compact, power-efficient hardware suitable for field-station deployment. Since LGH and the auxiliary motion head are training-only components, the deployed model has an identical computational cost to the single-task baseline.

**Figure 5:**
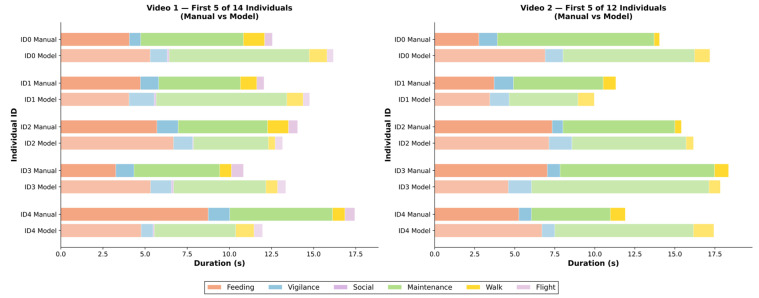
Comparison of Individual-Level Behavior Time Distribution Between Manual Annotation and Model Prediction.

Conventional human observation is limited by viewing windows and selective attention, typically recording only the dominant group behaviors or key episodes of a few individuals. In contrast, AviaTAD-LGH provides frame-level, individual-level behavior detections, enabling the reconstruction of the complete behavioral time series for each bird. Figure 5 illustrates the time-budget patterns of all individuals in the two videos.

For Video 1, manual annotations for individual ID1 show that it primarily engages in feeding (4.7 s) and maintenance (4.8 s), totaling 12.1 s. However, the model reveals a more detailed behavioral profile: 4.0 s of feeding, 1.5 s of vigilance, 7.8 s of maintenance, 1.0 s of walking, and 0.1 s of social behavior, totaling 14.8 s—22.4% longer than the manually recorded duration. Similar patterns are observed across ID0–ID4 in both videos, demonstrating that the model captures short-lived behavioral transitions and secondary activities that are often overlooked in traditional observation.

Figure 5 compares individual-level behavior time budgets between manual annotation and model prediction. The left panel shows the first 5 of 14 individuals in Video 1, and the right panel shows the first 5 of 12 individuals in Video 2 (selected for readability; full data in Supplementary Material). Each individual is represented by two rows: Manual (human labels) and Model (AviaTAD-LGH predictions). The colors of the stacked bars correspond to different behavior categories. Overall, the model tends to produce longer total behavior durations and more fragmented behavior sequences, reflecting the advantage of frame-level detection in terms of temporal resolution.

Access to individual-level data enables more fine-grained ecological analyses. For instance, by comparing the proportion of time spent feeding among individuals within the same flock, one can infer social hierarchies or the intensity of resource competition. Examining vigilance time across group members (e.g., individual ID1 in Video 2 spends 1.2 s in vigilance, slightly above the group mean of 0.9 s among the displayed individuals) can help identify sentinel individuals and study the division of labor in anti-predator strategies. Such data granularity is difficult to achieve with traditional methods due to high labor costs, but the automated nature of AviaTAD-LGH enables long-term, high-frequency tracking of individual behavior.

**Figure 6:**
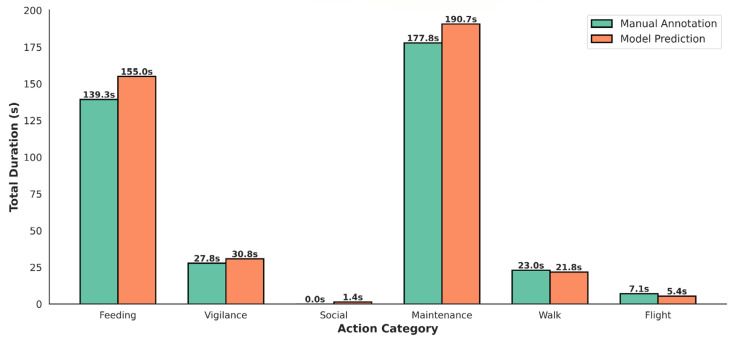
Comparison of the overall distribution of behavior durations.

In terms of behavioral coverage, model predictions substantially outperform manual annotation. As shown in Figure 6, AviaTAD-LGH detects a total of 405.0 s of behavioral activity across the two videos, compared with 375.0 s from human annotation—an 8.0% increase. This difference mainly arises from two factors: (1) the model captures sub-second behavioral transitions, such as rapid head-turns during vigilance; and (2) AviaTAD-LGH is better at decomposing overlapping behaviors, for example, identifying head movements during feeding as separate vigilance segments. At the category level, agreement between the model and human annotations varies. For feeding, the model predicts 155.0 s, compared to 139.3 s from manual labels (+11.3%), with an average similarity of 89.5%. For maintenance, AviaTAD-LGH predicts 190.8 s versus 177.8 s (+7.3%), with 92.7% similarity. For vigilance, the model yields 30.7 s compared to 27.8 s (+10.4%), with 89.9% similarity. These three high-frequency behaviors all show similarity scores around 90%, supporting the reliability of AviaTAD-LGH for the dominant activity types. In contrast, dynamic behaviors such as walking (67.9% similarity) and flight (67.6%) exhibit lower agreement, reflecting the inherent difficulty of boundary determination under fast motion.

### 4.4. Optimization Analysis of LGH

To evaluate the effectiveness of the proposed LGH strategy in mitigating gradient conflicts during multi-task learning, we conduct a systematic analysis of the optimization dynamics.

**Figure 7:**
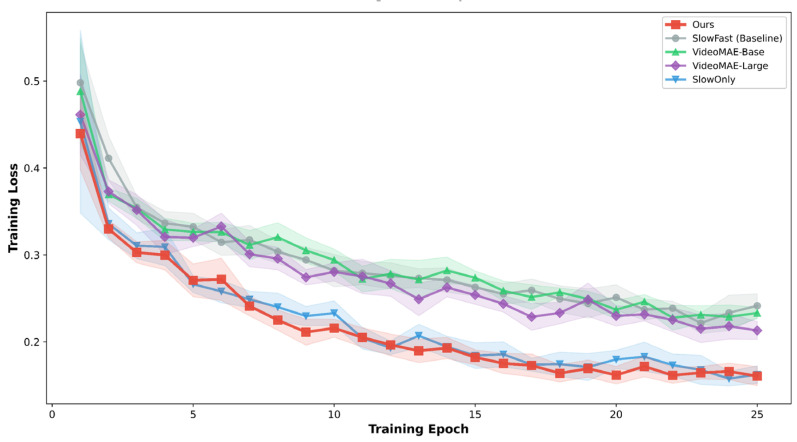
Training loss curves.

Figure 7 presents the training loss curves for five model configurations over 25 epochs, including four models integrated with the proposed LGH strategy and one SlowFast baseline without LGH for comparison. Our method achieves the lowest final loss of 0.1605, representing a 63.5% reduction from the initial value of 0.4396. Compared with the baseline (without LGH), which converges to a final loss of 0.2415 (45.1% reduction), our approach yields a substantial improvement of 18.4 percentage points in loss reduction. Furthermore, our method demonstrates accelerated convergence, reaching the 0.3 loss threshold at epoch 4 and the 0.2 threshold at epoch 12, whereas the baseline requires epochs 9 and 18, respectively, and fails to reach the 0.2 threshold entirely. Among other backbones, SlowOnly exhibits comparable convergence behavior (final loss: 0.1624, 63.1% reduction), while VideoMAE-Large and VideoMAE-Base converge to higher final losses of 0.2131 and 0.2332. The narrow 95% confidence intervals across all models indicate stable training dynamics. These results validate that LGH effectively accelerates optimization and improves convergence when integrated with the SlowFast architecture.

**Figure 8:**
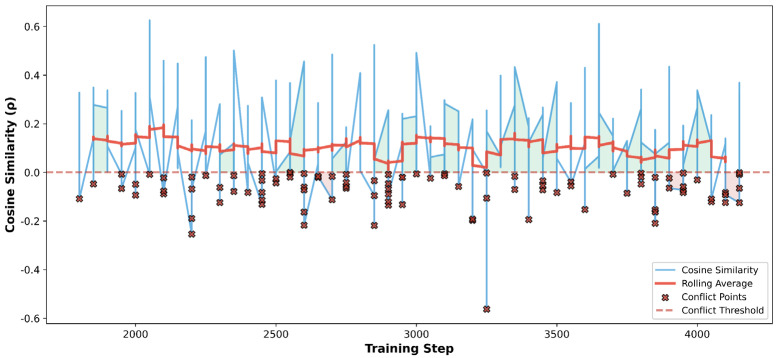
Gradient cosine similarity evolution.

Figure 8 visualizes the cosine similarity ρ between the primary (action classification) and auxiliary (motion attribute) task gradients throughout the LGH-Adaptive phase (steps 1750–4250). The instantaneous cosine similarity (blue curve) exhibits substantial fluctuation, ranging from approximately −0.55 to +0.62, indicating that gradient conflicts occur frequently during training. However, the rolling average (red curve) remains consistently positive, stabilizing within the range of 0.05–0.15 throughout the optimization process. This behavior demonstrates that while transient gradient conflicts are inevitable in multi-task learning, LGH successfully maintains an overall cooperative optimization direction by adaptively down-weighting the auxiliary loss when gradients temporarily disagree. The sustained positivity of the rolling average confirms that the auxiliary motion branch provides complementary kinematic information for action recognition in the majority of training iterations, and that LGH prevents accumulated interference from episodic conflicts, thereby ensuring stable convergence of the primary task.

**Figure 9:**
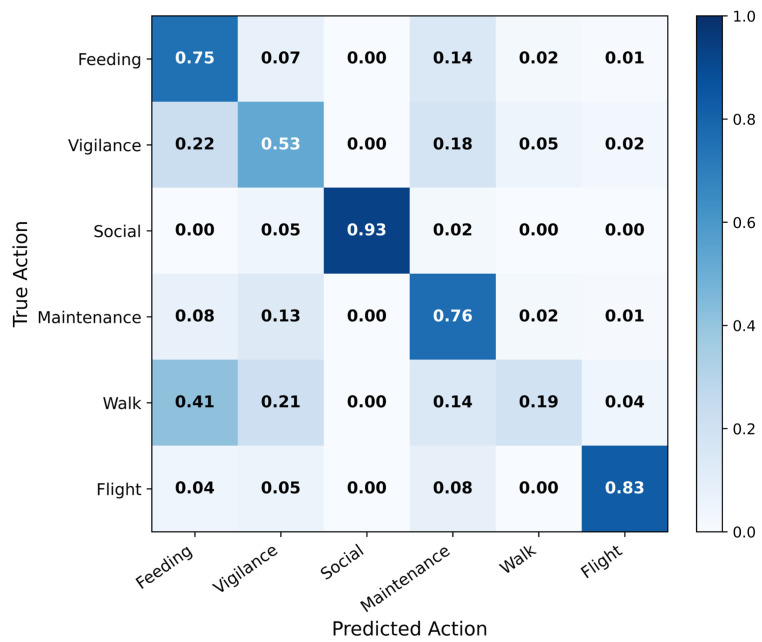
Normalized confusion matrix of AviaTAD-LGH on the test set.

To further analyze classification patterns, Figure 9 presents the normalized confusion matrix of AviaTAD-LGH on the test set. The dominant diagonal entries confirm strong classification accuracy for social behavior (0.93), flight (0.83), maintenance (0.76), and feeding (0.75). The main off-diagonal confusion patterns are ecologically interpretable: vigilance is most frequently confused with feeding (0.22) and maintenance (0.18), reflecting the visual similarity between brief head-raising during foraging and the still postures shared by these three behaviors. Walking is the most challenging class, with 41% of instances misclassified as feeding and 21% as vigilance, due to the inherent ambiguity of slow locomotion with intermittent pecking in field conditions. Feeding shows its largest confusion with maintenance (0.14), where similar stationary body postures with different head and neck movements are difficult to distinguish. The contrast between walking (AP 23.20%) and flight (AP 85.95%)—both dynamic behaviors—is notable: flight involves large-scale displacement and distinctive wing movements that are visually unambiguous, whereas walking often co-occurs with feeding postures and differs only in subtle leg movement frequently occluded by vegetation or water.

### 4.5. Daily Activity Rhythms of the Hooded Crane

**Figure 10:**
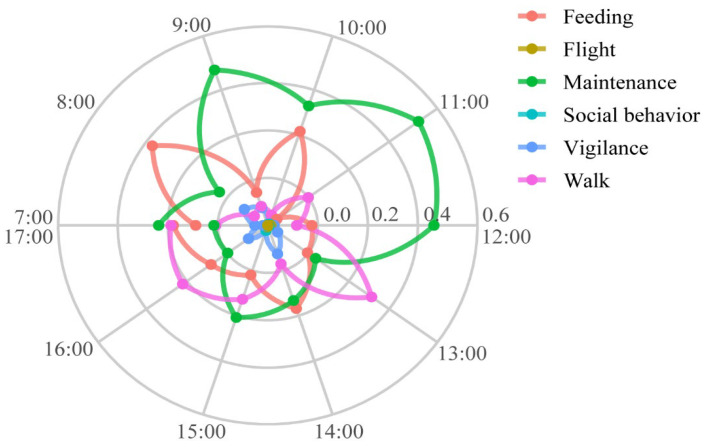
Diurnal Activity of the Hooded Crane.

Based on observational data collected from the Hooded Crane at the Chongming Dongtan National Nature Reserve, Shanghai, on 9 November 2025, between 7:00 A.M. and 6:00 P.M., we analyzed the temporal distribution of the crane’s behavior across different time intervals throughout the day. To eliminate the influence of absolute value differences, behavior data were standardized into relative proportions on an hourly basis. The time distribution characteristics were visualized using polar coordinate continuous curves, providing an intuitive representation of the crane’s daily activity dynamics. The results revealed significant differences in the behavior of the Hooded Crane throughout the day (Figure 10). Feeding behavior rapidly increased and reached its peak between 8:00 A.M. and 9:00 A.M., then remained elevated until gradually declining towards noon. After 1:00 P.M., feeding activity increased again, but the peak was lower than the morning’s, indicating that the morning was the main feeding period. Maintenance behavior showed considerable fluctuation throughout the day, with a higher proportion in the early morning (7:00 A.M. to 8:00 A.M.), which decreased as feeding activity increased. Maintenance again rose during the periods of 11:00 A.M. to 12:00 P.M. and 2:00 P.M. to 3:00 P.M., before stabilizing in the evening. Walking behavior exhibited relatively stable fluctuations, but it was enhanced during periods of reduced feeding and increased maintenance, reflecting the spatial movement process between active states. Vigilance behavior remained relatively low overall but showed slight time-dependent variation, decreasing during the peak feeding periods and increasing during rest or low-activity periods. Flight behavior remained at minimal levels across most of the day, indicating that the Hooded Crane primarily engaged in ground-based activities during this observation period. Social behavior also maintained a low proportion, with a slight increase in the afternoon, possibly corresponding to interactions between individuals or adjustments in position. In summary, this analysis of daily activity rhythms not only revealed the clear diurnal activity patterns of the Hooded Crane in the wetland ecosystem but also validated the effectiveness of the AviaTAD-LGH model in identifying and quantifying bird behavior dynamics in natural settings.

## 5. Discussion

Taken together, the comparative experiments and ablation studies demonstrate that AviaTAD-LGH provides consistent performance improvements on our crane-behaviour dataset. Under matched backbones and training settings, AviaTAD-LGH improves overall mAP by 2.80 percentage points relative to the single-task baseline and yields substantial AP gains for challenging behaviours such as vigilance and walking. These results indicate that the proposed multi-task formulation and the associated optimization strategy contribute beyond changes in model capacity.

A central motivation of this work is to support scalable ecological monitoring from continuous ground-based video sensing, which can be viewed as a form of close-range observation complementing conventional remote sensing products. The comparison with manual annotation shows that AviaTAD-LGH offers practical advantages in three aspects: processing efficiency, behavioural coverage, and individual-level granularity. On two representative field-monitoring videos, the system achieves a 35.4× average efficiency ratio, recovers an additional 8.0% of accumulated behavioural duration, and produces time-budget distributions that are highly consistent with manual labels for dominant behaviours. Importantly, AviaTAD-LGH converts long surveillance streams into time-stamped behavioural products—including per-individual event logs and time budgets—that can be consumed by downstream ecological analyses and monitoring workflows.

From a modelling perspective, AviaTAD-LGH enhances fine-grained behaviour recognition through joint learning of semantic behaviours and a binary static/dynamic motion attribute. The auxiliary motion attribute provides an additional kinematic cue that complements visual semantics and is particularly helpful for behaviour pairs that are visually similar but differ in movement patterns (e.g., walking vs. feeding, vigilance vs. maintenance). Under limited and long-tailed field datasets, this multi-task formulation also acts as a form of inductive bias and regularization, improving robustness across diverse scenes and recording conditions.

A key challenge in multi-task learning is negative transfer caused by conflicting gradient directions between tasks. LGH addresses this problem by adapting task loss weights using the cosine similarity between task gradients, suppressing conflicts without modifying the network architecture or optimizer. The observed gradient-similarity statistics and training-loss dynamics are consistent with the role of LGH in stabilizing optimization, thereby enabling the auxiliary motion task to contribute positively to the primary behaviour detection task. Nevertheless, LGH currently uses a simple weighting mechanism, and future work may explore continuous weighting functions or multi-objective formulations to achieve finer task balancing and to further improve stability under more heterogeneous data.

From an ecological monitoring standpoint, it is important to understand where the system is reliable and where uncertainty remains. AviaTAD-LGH outputs are more stable for dominant, longer-duration behaviours, whereas agreement is lower for fast dynamic behaviours (e.g., walking and flight), where temporal boundaries are inherently ambiguous under rapid motion, occlusion, and motion blur. These limitations are typical for continuous video understanding in complex field scenes and should be considered when deriving fine-grained indicators. In practice, uncertainty can be managed through targeted manual spot checks, confidence threshold calibration, and temporal smoothing or aggregation when computing behaviour budgets and activity profiles.

The individual-level time series produced by AviaTAD-LGH further extends the utility of ground-based video sensing. Compared with traditional observation, automated frame-level detection enables high-temporal-resolution behavioural trajectories for each individual, revealing short transitions and secondary events that are easily missed by human observers. Such trajectories support analyses of within-flock heterogeneity (e.g., individual variation in feeding allocation, vigilance contribution, and interaction frequency) and facilitate long-term, high-frequency monitoring at a feasible labour cost.

More broadly, AviaTAD-LGH provides a generalizable pipeline that bridges spatio-temporal action detection with the extraction of behavioural indicators relevant to ecological assessment. While this study focuses on cranes and includes wetland scenes, the framework is not restricted to a specific species or habitat type: given an appropriate behaviour taxonomy and spatially annotated video data, the same training and deployment pipeline can be transferred to other wildlife monitoring scenarios. In a multi-scale environmental monitoring context, these behavioural products can complement satellite or UAV-derived habitat covariates by providing fine temporal detail at key sites, thereby supporting integrated analyses of habitat dynamics and animal responses.

## 6. Conclusions

In this work, we present AviaTAD-LGH, a multi-task system for spatio-temporal action detection of wild-bird behaviours in continuous field videos. By jointly modelling behaviour categories and a static/dynamic motion attribute, AviaTAD-LGH enriches the learned representation for fine-grained discrimination, while LGH-based gradient coordination mitigates negative transfer during multi-task optimization. On our custom crane-behaviour dataset, AviaTAD-LGH achieves 68.60% mAP, outperforming a strong single-task baseline. Ablation studies and gradient analyses further demonstrate the effectiveness of LGH and related design choices for stabilizing multi-task training and improving performance.

As a practical monitoring pipeline, AviaTAD-LGH enables real-time, individual-level behaviour extraction from ground-based video sensing streams and provides interpretable outputs such as event logs and time budgets. These behavioural products can support scalable ecological monitoring and facilitate habitat assessment analyses in field settings, reducing reliance on labour-intensive manual annotation.

## Figures and Tables

**Table 1:** Classification of six bird behavior types.

Behavior Type	Behavior Description
Feeding	Behaviors such as lowering the head to search, probing, pecking, digging with the bill, swallowing and other food-handling movements, drinking, adults feeding chicks, and chicks begging food from adults.
Vigilance	behaviors such as stretching the neck, scanning the surroundings and gazing into the distance.
Social behavior	behaviors such as chasing among conspecifics, threatening or responsive calls, flights immediately before or after obvious provocative acts, and courtship behavior.
Maintenance	behaviors such as preening, shaking the plumage, wing flapping, resting and bathing.
Walk	walking movements not associated with feeding and not accompanied by provocative or aggressive behavior.
Flight	flying.

**Table 2:** Performance comparison of different models on the bird behavior dataset.

Model Name	Overall mAP	Gain over Baseline	Gain over Original	Feeding	Vigilance	Social	Maintenance	Walk	Flight
Original models (without LGH)									
VideoMAE-Base (baseline)	0.6116	0%	-	0.6315	0.4638	0.9502	0.6792	0.1071	0.8377
ACRNN-SlowFast	0.6143	+0.27%	-	0.8117	0.5099	0.8233	0.7127	0.1673	0.6607
SlowOnly	0.6255	+1.39%	-	0.8488	0.5115	0.9345	0.7155	0.1337	0.6088
VideoMAE-Large	0.6518	+4.02%	-	0.7510	0.4980	0.9161	0.6680	0.1614	0.9166
SlowFast	0.6580	+4.64%	-	0.8148	0.4778	0.9617	0.6934	0.2199	0.7806
Multi-task learning models (+LGH)									
ACRNN-SlowFast +LGH	0.6229	+1.13%	+0.86%	0.7020	0.4386	0.8535	0.6690	0.1983	0.8763
VideoMAE-Base +LGH	0.6333	+2.17%	+2.17%	0.7235	0.4594	0.9353	0.6530	0.1405	0.8881
SlowOnly +LGH	0.6717	+6.01%	+4.62%	0.8236	0.4940	0.9344	0.7076	0.2015	0.8690
VideoMAE-Large +LGH	0.6760	+6.44%	+2.42%	0.8534	0.5618	0.9241	0.6399	0.1622	0.9147
AviaTAD-LGH (Ours)	0.6860	+7.44%	+2.80%	0.8318	0.5477	0.9403	0.7046	0.2320	0.8595

**Table 3:** Ablation study results (mAP).

Configuration	mAP	Compared with Baseline
Single-task baseline	65.80	-
+GradNorm	66.21	+0.41%
+PCGrad	66.92	+1.12%
+RI-PCGrad	67.25	+1.45%
+LGH	68.60	+2.80%

**Table 4:** Component ablation of the auxiliary motion head and weighting strategy (mAP).

Configuration	Aux. Motion Head	Weighting Strategy	mAP	Over Baseline (pp)
Single-task baseline	✗	—	65.80	-
Motion head	✓	equal sum (λ = 1.0)	66.01	+0.21
Motion head + static weighting	✓	fixed λ = 0.5	67.20	+1.40
Motion head + uncertainty weighting	✓	uncertainty-based	67.76	+1.96
Motion head + LGH (Ours)	✓	conflict-aware reweighting	68.60	+2.80

**Table 5:** Hyperparameter sensitivity analysis of LGH (mAP, %).

	λ_base = 0.02	λ_base = 0.05	λ_base = 0.10	λ_base = 0.20
α = 0.1	67.23	67.81	68.14	67.52
α = 0.2	67.41	67.95	68.60	67.73
α = 0.3	67.18	67.86	68.26	67.44
α = 0.5	67.01	67.53	67.89	67.12

**Table 6:** Model complexity and inference speed comparison.

Model	Params (M)	FLOPs (G)	Inference Speed (fps)
VideoMAE-Base	86.6	180.1	38.5
VideoMAE-Large	304.4	597.2	14.2
ACRNN-SlowFast	35.8	71.3	45.6
SlowOnly	31.7	54.5	52.3
SlowFast	33.6	65.7	48.1
AviaTAD-LGH (Ours)	33.6	65.7	48.1

## Data Availability

The original contributions presented in this study are included in the article. Due to ongoing research and restrictions related to the data collection sites, the dataset is available from the corresponding author upon reasonable request. Further inquiries can be directed to the corresponding author.

## Supplementary Materials

The supporting information can be downloaded at https://www.mdpi.com/article/10.3390/s26072088/s1.

## Abbreviations

The following abbreviations are used in this manuscript:

AviaTAD-LGH	Avian Temporal Action Detector with Lightweight Gradient Harmonization
LGH	Lightweight Gradient Harmonization
AP	Average Precision
mAP	Mean Average Precision
ROI	Region of Interest
SGD	Stochastic Gradient Descent
